# Retroperitoneal Lipomatosis

**DOI:** 10.5334/jbsr.4062

**Published:** 2025-10-25

**Authors:** Floriane Bootsma, Adelard De Backer

**Affiliations:** 1 Vrije Universiteit Brussel (VUB), Universitair Ziekenhuis Brussel (UZ Brussel), Department of Radiology, Laarbeeklaan 101, 1090 Brussels, Belgium

**Keywords:** CT, retroperitoneal lipomatosis

## Abstract

*Teaching point:* Retroperitoneal lipomatosis is a benign idiopathic process resulting in a progressive, symmetrical and homogeneous increased retroperitoneal fat lesion without areas of increased density.

## Case History

A 71-year-old woman presented with vague, non-specific, diffuse abdominal pain and gradual abdominal distension for several years. Clinical examination of the abdomen confirmed abdominal distension without rebound tenderness and revealed a diffuse lumpy feel and a generalized dull note on percussion. The patient was referred for contrast-enhanced computed tomography (CT) to exclude a diffuse abdominal lump or loculated ascites.

**Figure 1:**
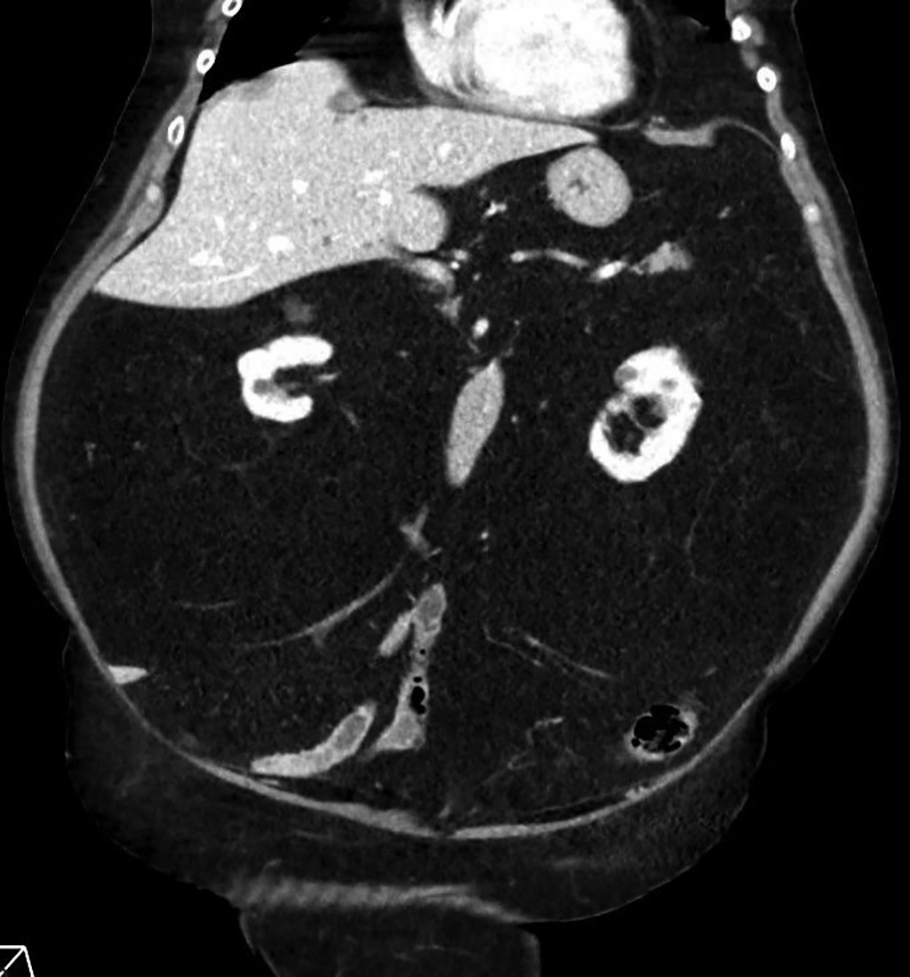
Coronal image demonstrates symmetrical and homogeneous increased retroperitoneal fat.

On CT, a non-capsulated low-density retroperitoneal lesion with diffuse and symmetric distribution was noted. No areas of increased density or contrast-enhancement were noted. An extensive increased amount of perirenal fat extended into the pelvis and resulted in the displacement of the anterior and posterior renal fascia and a decrease of the peritoneal space (Figures 1, 2a and b, and 3). Hydronephrosis resulting from ureteral compression was absent. Based on CT findings, a diagnosis of diffuse retroperitoneal lipomatosis (RL) was made.

**Figure 2a:**
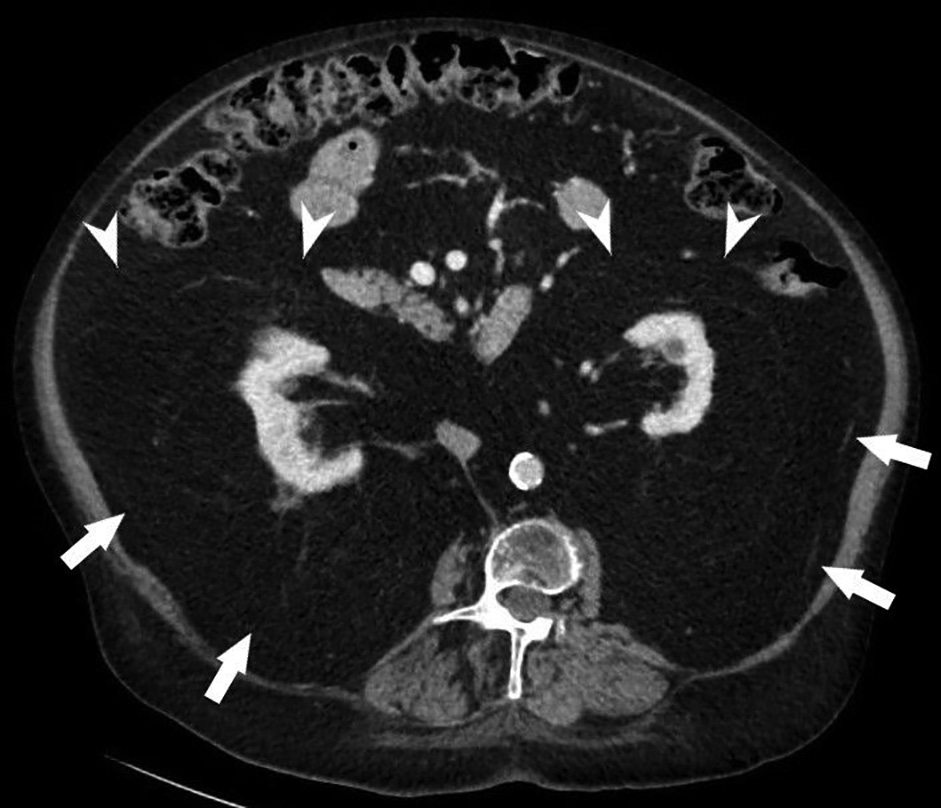
RL with displacement of the anterior (arrowhead) and posterior renal fascia (arrow).

**Figure 2b:**
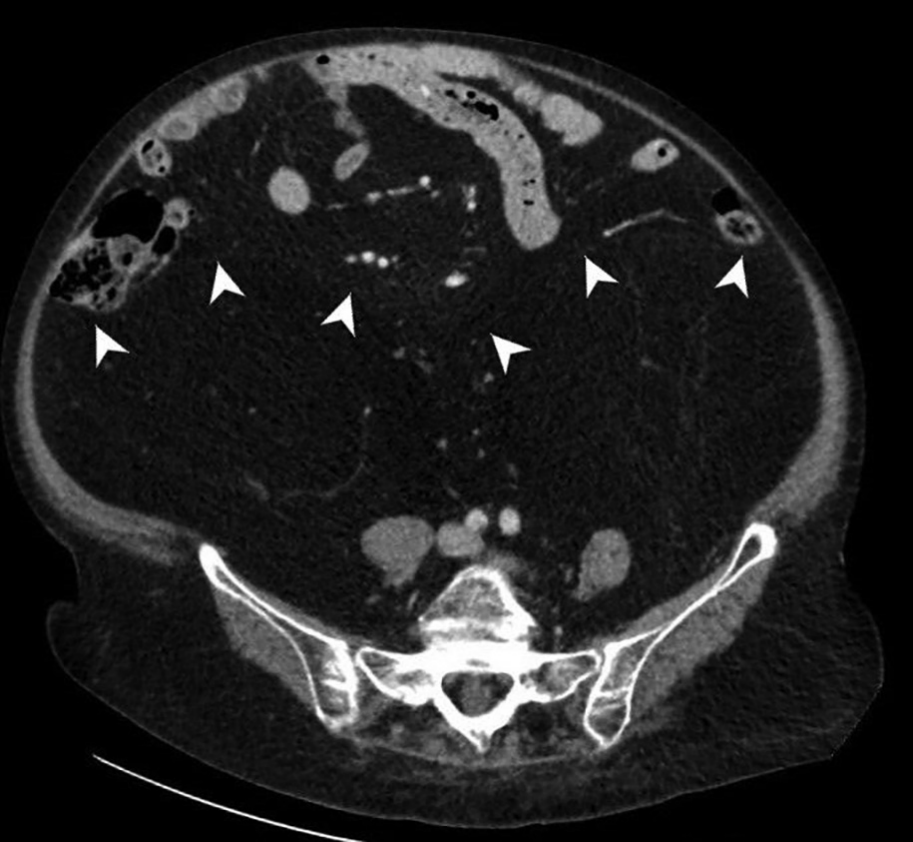
CT shows symmetrical and homogeneous increased retroperitoneal fat.

**Figure 3:**
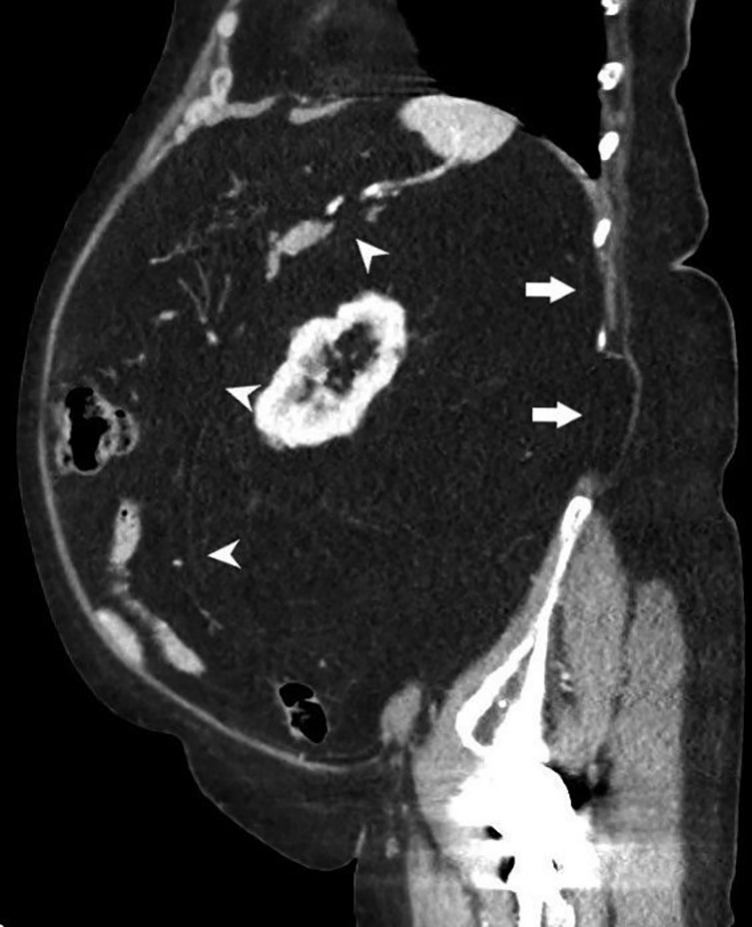
Sagittal image showing RL resulting in decrease of the peritoneal space.

## Comments

RL is a benign condition resulting from a non-encapsulated overgrowth of mature fat tissue in the retroperitoneal space. The incident of RL has been reported at around 1.7 cases per 100,000 population with a black male predominance [1]. RL is often associated with an increased amount of symmetrically distributed fat elsewhere in the body, for example in the subcutaneous tissues of the cervical, thoracic, abdominal and pelvic regions. It may also be found in the deltoid muscle. This condition is known as multiple symmetric lipomatosis.

Patients may be asymptomatic or present with non-specific complaints such as abdominal fullness, discomfort or vague pain. Urinary or gastrointestinal complaints are often present.

Ultrasound may show diffusely increased retroperitoneal fat, seen as homogeneous hyperechoic, with encasement of retroperitoneal organs and anterior displacement of abdominal organs. Extensive pelvic involvement with extrinsic bladder compression may result in a tubular shape.

CT and magnetic resonance imaging (MRI) are the preferred imaging methods for diagnosis. On CT, a symmetrically and homogeneously fat-attenuating retroperitoneal lesion without areas of increased density or contrast enhancement is seen. However, a few thin non-enhancing septa may be noted within the lesion. Mass effect may result in a variable degree of displacement of intraperitoneal organs, compression on ureters and deformation of the bladder. On MRI, the lesion is hyperintense on T1- and T2-weighted images with complete suppression of signal intensity on fat-suppressed images.

The differential diagnosis includes well-differentiated liposarcoma, lipoma, hibernoma, teratoma and myelolipoma. Patients with retroperitoneal liposarcoma or lipoma may also have increased amounts of fat in the retroperitoneal and intraperitoneal spaces. When a biopsy is performed to differentiate between RL, lipoma and liposarcoma specific sampling, including karyotyping, is required.

There is no definite treatment for RL. Mildly symptomatic patients are treated conservatively. Urinary outflow obstruction with impaired renal function may require stenting of the obstructed ureters or surgery with fat excision to relieve the mass effect.
